# Future Spaces: Reinventing the Home Network for Better Security and Automation in the IoT Era

**DOI:** 10.3390/s18092986

**Published:** 2018-09-07

**Authors:** Mathieu Boussard, Dinh Thai Bui, Richard Douville, Pascal Justen, Nicolas Le Sauze, Pierre Peloso, Frederik Vandeputte, Vincent Verdot

**Affiliations:** 1 Nokia Bell Labs, 1 route de Villejust, 91620 Nozay, France; mathieu.boussard@nokia-bell-labs.com (M.B.); dinh_thai.bui@nokia-bell-labs.com (D.T.B.); richard.douville@nokia-bell-labs.com (R.D.); pierre.peloso@nokia-bell-labs.com (P.P.); Vincent.verdot@nokia-bell-labs.com (V.V.); 2 Nokia Bell Labs, Copernicuslaan 50, 2018 Antwerp, Belgium; pascal.justen@nokia-bell-labs.com (P.J.); frederik.vandeputte@nokia-bell-labs.com (F.V.)

**Keywords:** smart home, Internet of Things, SDN, NFV

## Abstract

Cyber-Physical Systems (CPSs) are complex systems comprising computation, physical, and networking assets. Used in various domains such as manufacturing, agriculture, vehicles, etc., they blend the control of the virtual and physical worlds. Smart homes are a peculiar type of CPS where the local networking fundamentals have seen little evolution in the past decades, while the context in which home networks operate has drastically evolved. With the advent of the Internet of Things (IoT), the number and diversity of devices connected to our home networks are exploding. Some of those devices are poorly secured and put users’ data privacy and security at risk. At the same time, administrating a home network has remained a tedious chore, requiring skills from un-savvy users. We present Future Spaces, an end-to-end hardware-software prototype providing fine-grained control over IoT connectivity to enable easy and secure management of smart homes. Relying on Software-Defined Networking-enabled home gateways and the virtualization of network functions in the cloud, we achieve advanced networking security and automation through the definition of isolated, usage-oriented slices. This disrupts how users discover, control and share their connected assets across multiple domains, smoothly adapting to various usage contexts.

## 1. Introduction

Homes have always constituted a peculiar context for technology adoption, even more so recently in the domain of information and communication technologies [1]. Among the differentiating characteristics, one can list the lack of users’ administration knowledge, usability constraints, security and privacy concerns, and the concurrent use by multiple users sharing the system with potentially diverging goals. The last few years have been marked by a wide availability of home networks served by broadband internet connections, complemented more recently with the development of “smart” consumer devices. This has given rise to practical embodiments of the long prophesized “smart home”, i.e., homes that provide advanced automation and delivery of services mixing the physical and digital worlds. The smart home infrastructure, composed of its underlying network and sensing or actuating devices, is an open-ended cyber-physical system (CPS), over which different smart home applications can be instantiated. The rise in the number of Internet of Things (IoT) devices is a key enabler of this vision, but it also presents a number of hurdles such as interoperability, usability, security and privacy issues, and more generally the lack of means for users to control what happens in their connected homes.

Indeed, it has recently become clear that a number of “things” in the Internet of Things are both insufficiently secured and pose serious concerns to user privacy. In parallel, interconnecting things from different homes remains a tedious process due to the current design of home networks. Indeed, the IoT-enabled smart home remains grounded on the same old Local Area Network (LAN) paradigm defined in the 1970s for small networks of static, trusted and often local computing devices, which did not rely on the Internet for operation. The resulting user experience is at best disappointing, while the aforementioned concerns over security and privacy threaten to deter wider adoption.

In this paper, we present an end-to-end research solution prototype called Future Spaces, which aims precisely at giving users simple means to control how their devices and home networks are used, relying on recent advances in networking technology–namely Software-Defined Networking (SDN) and Network Function Virtualization (NFV). The solution allows users to dynamically control their digital assets. Secure access to devices is enforced through fine-grained communication across groups of resources on dedicated, dynamic and isolated virtual networks, or “slices”. This supports the vision of a worry-free cyber-physical world, where people can intuitively and securely organize, trust and share their IoT devices, content and services. This vision is realized thanks to the following core aspects:

- “Virtual Places”, realizing the foundation of new smart home/environment CPS by logically representing and managing an administrative domain, voluntarily blurring the frontier between the physical and virtual environments to cope with new usages and needs of end-users. Challenges in realizing such a concept lie in the definition and implementation of the companion cloud infrastructure and network gateways, realizing a distributed testbed allowing to easily provision, deploy, dynamically extend to multiple geographical locations, and interconnect these Virtual Places;
- “Virtual Spaces”, allowing one to create secured, dynamic collections of resources regrouped by usage, and interconnecting these resources over one or more Virtual Places (i.e., crossing multiple administrative home domains). Realizing those Virtual Spaces requires on the one hand a mix of mechanisms to automatically adapt their content according to their definition and the current context. On the other hand, the historical control of Local Area Networks is revisited by using dynamically programmable “Software-Defined LANs” enabling the connection of the resources they regroup into isolated network slices, while ensuring network-based security in open and living environments;
- Simple tools and interfaces together with advanced automation mechanisms, allowing users to exercise better control over their digital assets and ultimately share them with others. Those mechanisms should act as a “digital butler” of future smart homes, reacting to orders from users, but also quietly enforcing users’ goals, privacy and security over their digital belongings.

Deriving from the definition and realization of these core aspects, our contributions are therefore as follows. First, we developed the supporting software/hardware management and networking infrastructure; it comprises cloud and network components to instantiate Virtual Places, supporting discovery and network mobility of resources across domains. Second, we implemented the domain controller software component that we call “MajordHome”, in charge of managing the users, resources and Virtual Spaces of a given Virtual Place, either through simple user interactions or automatically on behalf of the user through a number of automation mechanisms. The latter range from adaptive Virtual Space definition, to the definition and enforcement of personal policies expressed using a domain specific language to protect user security and privacy, to the broader support of third-party defined logic through Assistants. Third, we implemented the dedicated network controller to realize LAN-like network slices (Software-Defined LANs), allowing a single-network-interface unmodified IoT device to be part of several, possibly cross-domains, network slices. Fourth, we validated the proposed approach through the provisioning of a distributed testbed orchestrating the different pieces of the solution, and the realization of a number of use case scenarios.

In the rest of the paper, we detail the key components of our solution and the realization of the proof of concept (PoC) prototype that orchestrates them. We first discuss the relevant state of the art in Section 2 and present an overview of the Future Spaces key concepts, components and working assumptions in Section 3. Section 4 describes the overall system architecture of our proof of concept implementation and related design choices. Section 5 details Software-Defined LANs realization and the dedicated network controller implementation. Section 6 depicts the MajordHome component focusing on user support and automation. Section 7 details on the testbed putting our proof of concept realization at work and an accompanying illustrative demonstration use case. Section 8 provides a discussion of the strengths and limitations of our solution before concluding.

## 2. Context and Related Work

### 2.1. The State of the Smart Home

Though the connected smart home has been an active research domain for many years, it has recently gained a lot of attention from the industry with the proliferation of smart objects and related IoT technology, leading to the apparition of a wealth of new players in the domain. Research and commercial versions of smart homes, e.g., domestic environments with sensing, actuation, and networked devices, have long been built, including Mozer’s adaptive house [2], Georgia Tech Aware Home [3], Orange [4], eHome [5], and MIT’s Housen [6]. As pointed by Brush et al. [7], although the term “smart home” referring to a home environment adapting to its inhabitants has caught the attention of the media and researchers, the term “home automation” defined as the capability to automate and control multiple disparate systems is a better description of currently available technology. Brought by the advent of affordable technology (e.g., low power networking such as Zigbee or Bluetooth LE, and network and computing on a chip), new types of smart devices pop up every day, targeted an expanding range of application domains, including user welfare (e.g., weight scales), security and information related monitoring (e.g., weather stations), as well as home automation (e.g., thermostats or smart locks), brown goods, etc. It is worth noticing however that the degree of smartness of these connected objects varies greatly, and that most of them operate independently of all other smart devices, or at best within a very limited (and often closed) ecosystem. This fosters the advent of multi-technology orchestration gateways and software, and more recently of voice controlled assistant services and accompanying devices. However, solutions focusing first on control of appliances and interoperability issues often suffer from business viability or strategic reviews (e.g., the Revolv hub was discontinued in 2016, two years after it was bought by Google) as well as from more fundamental challenges.

Back in 2001, Edwards and Grinter [8] from PARC listed the seven challenges to be tackled before smart homes can become a reality. The first one is dubbed the “accidentally smart home”, i.e., the fact that technology may be added in a “piecemeal” fashion to the home (contrary to the testbeds mentioned earlier which were designed from the ground up). Besides technical considerations (“impromptu interoperability” caused by incremental and/or dynamic addition of components to the smart home), the authors point to the problem of the intelligibility of such complex, dynamic systems for the user, who is often the only available administrator, and often with limited skills for such tasks, as pointed in another identified challenge. Other challenges listed include: taking into account the specificities of the domestic environment, coping with the social implications of smart home technology (e.g., on privacy), reliability, as well as determining (and applying!) the right amount of ambient intelligence such that the system is “predictable, intelligible, and recoverable”. Interestingly, several of these challenges still hold nearly two decades later, as pointed in [9] where the author immersed herself in a 2018 smart home embodiment based on available IoT devices and orchestration logic: while the expected privacy concerns endure, the resulting user experience suffers from limited usability, and even limited usefulness!

### 2.2. Relevant Related Work on Home Networking and Security

While the full-fledged smart home vision is still not realized, smart domestic appliances have multiplied and home networking as their serving infrastructure has received growing attention from the scientific and technical community. The specificity of home networks (e.g., lack of a knowledgeable administrator, usability constraints, privacy concerns, etc. [10]) and the growing concerns over the limited security of smart appliances (both regarding devices themselves, mostly for economic reasons as pointed out in [11], as well as middleware or orchestration frameworks [12]) have led to many research and commercial proposals.

Among recent trends in smart homes, the assessment and control of security and privacy risks has been one of the hottest areas investigated by researchers and innovative solutions. As an example, [13] proposes a new research trajectory that consists in applying a four-step cycle “Scenario-Test-Evaluate-Propose” to smart home IoT devices. The first step consists in creating under laboratory conditions realistic scenarios in which people are likely to use IoT devices at home. Next, devices identified for the previous scenarios are tested against four (security and privacy) criteria namely confidentiality, integrity, access control and reflection. Test results are then “evaluated” by different home IoT actors (e.g., suppliers, consumers, insurers, regulators) gathered together during a workshop. According to their reactions and expectations, proposals can finally be made to mitigate identified security and privacy concerns (e.g., security star rating, build security at design stage). Concerning network control to enhance security, a number of propositions have looked at providing on demand and dedicated WiFi networks (SSIDs) for different users of the home (e.g., parents/administrators, children, visitors), or constraining which devices they can access and for how long and controlling their access to the Internet. This highlights the need for tighter network control compared to the traditional home Local Area Network (LAN) management, in particular providing fine-grain control of packet forwarding at the access point according to the privileges granted per user-group. For example, the commercial HomePass solution from Plume WiFi [14] allows to define personalized access rights to the home network and its connected devices. Going further on the control of device connectivity, Cujo [15] proposes a smart firewall relying on machine learning to help users to identify connected devices and alert them upon detection of abnormal behaviors. Access control and monitoring mechanisms relying on advanced programmable control points and augmented intelligence are therefore crucial properties for future smart home networks to address the growing concerns in terms of security and privacy while coping with the general lack of expertise of most end-users.

A recent trend in network automation is to rely on Software-Defined Networking (SDN). A key feature of SDN is the possibility to support network “slicing”, i.e., creating, on the same physical network infrastructure, a logical separation of traffic into isolated “slices” with differentiated characteristics. For example, FlowVisor [16] and GENI [17], proposed network virtualization mechanisms for campus and research networks to support different test beds along with production traffic on the same network infrastructure. Mimicking computing virtualization, they proposed a network hypervisor that enables the creation of several logical forwarding matrices or “flow spaces” (by analogy with the Virtual Machine concept) over the same forwarding hardware, isolated from each other with regards to multiple criteria such as bandwidth, CPU, forwarding tables, etc.

While SDN-based approaches have been proposed for enterprise and datacenter networks, their use in home and broader IoT environments is still being investigated [18]. Regarding the connected home, SDN allows to facilitate the home network management, service delivery and can be used to improve security. Slicing can be used to segregate traffic flows between users, and support differentiated characteristics (e.g., prioritization or more generally dedicated Quality of Service) or enforce data caps. Yiakoumis et al. [19] proposed to slice the home network infrastructure between multiple service providers (e.g., an energy provider, an Internet Service Provider (ISP), an over-the-top video provider, etc.) and users (via a guest network slice). Gharakheili et al. [20] propose an over-the-top solution allowing users to share the residential Internet connection while enforcing usage priorities and optimizing the user Quality of Experience. The solution consists of a user interface (web-based or mobile app) for management working alongside a modified open-sourced OpenFlow controller. Similarly, Kim et al. [21] proposed to control the usage of different persons at home based on user pre-defined cap usage rules. Other studies [22,23] proposed an SDN revisit of the home router to provide user-friendly management interfaces and services, such as providing a separate slice for guest devices, or supporting user-controlled device onboarding. The Future Spaces solution addresses these different aspects, by providing simple user controls and automation to pilot the home network through slicing. It goes further by supporting complex distributed and even cross-home scenarios, a feature not proposed by the state-of-the-art to our knowledge—especially for unmodified devices running no additional software (e.g., VPN client) or specific network configuration (e.g., VLAN), possibly assigned to multiple slices simultaneously.

Some approaches have focused on the security advantages of SDN in home environments through isolation slicing, fine-grained flow control and advanced monitoring and mitigation support [24,25] as well as the possibility to delegate the security of home networks to third parties thanks to programmability [26]. Through automation support, our approach supports the similar control and monitoring capabilities, while providing means for users to set sharing policies on their resources (see Section 6).

A companion to the SDN trend is that of Network Function Virtualization (NFV), and for home networks, the notion of a virtual Residential Home Gateway, where a number of network functions (e.g., NAT, DHCP, etc.) can be migrated from the home gateway device towards the ISP cloud. For ISPs, such an approach promises to lower the cost of maintaining fleets of heterogeneous device versions while facilitating the introduction of new services. In our solution, we rely on SDN and Network Functions Virtualization to redefine the home network, supporting dynamic and secure slicing to connect groups of selected devices within a single or across several households.

## 3. Overview of the Future Spaces Solution

### 3.1. Problem Statement and Working Assumptions

As stated in Section 1, our goal is to provide better control to the user, with easy-to-use concepts and tools to both facilitate usage and management of smart home digital assets, while drastically improving their security. To achieve this, we focus on the fine-grained control of the underlying network through group-based slicing, i.e., the definition of multiple independent virtual networks on top of the common home network infrastructure to interconnect groups of devices. The Future Spaces solution aims at supporting slices that are multi-location (i.e., where digital assets can be distributed geographically) and multi-user (i.e., shared between different users who desire to share their resources, with safeguards). To support commercial, off-the-shelf devices, our working assumption was that no specific software or networking configuration should be expected from the end devices. In parallel, to ensure that devices will work out of the box on our solution, typical local network communication (e.g., supporting multicast addressing for service discovery protocols) should be supported within the slices defined by the device groups, while one given device could pertain to multiple groups. These constraints led us to define a novel slicing mechanism named Software-Defined LAN, in which connectivity is constructed progressively between authorized devices (belonging to the same group, or Virtual Space), by adding forwarding rules for unicast and multicast traffic to network switching elements. SD-LANs mechanisms are further detailed in Section 5.

Finally, to ease its deployment on real networks, we have implemented our solution as an overlay on top of the ISP network. As a result, we focused mostly on the switching/routing control functionalities of the residential gateway, and not on the control of the ISP’s access link. Our prototype solution could therefore be deployed on any access point providing an Internet access and be operated by an over-the-top player running the Future Spaces infrastructure and components described in this paper. However, an implementation running as a fully ISP-managed solution would provide in addition better control of the access link to ensure end-to-end quality of service. Such an approach would typically be aligned with the virtualization of the home gateway trend at work in many ISPs today and bears similar operational constraints (moving to a cloud computing model as opposed to traditional network management) and advantages (improving network maintenance, simplifying and speeding deployment of new and innovative services...), while supporting advanced scenarios. Of course, a resource sharing service is even more relevant when widely accessible. Therefore, it is highly desirable that the deployed technical solution supporting this service be interoperable between ISPs. The Future Spaces end-to-end solution supports rich use cases through a set of essential features:

- *Fine-grained, context-aware access control*, achieved by providing a dynamic, fine-grained and group-based connectivity layer, allowing or disallowing communications between physical and/or digital assets. In our solution, by default no communication is allowed between devices attached to the Virtual Place network. By defining Virtual Spaces for groups of devices sharing the same user-defined context of use (e.g., multimedia consumption, home security), communication is only enabled within these groups of devices (through dedicated slices whitelisting communication), even though each device can be attached to geographically distant network elements;
- *Automatic security & privacy protection*, achieved by providing strong network isolation along with automatic policy enforcement, and protecting data privacy and network integrity of resources. The slices realizing the virtual spaces are isolated from each other in terms of data traffic, meaning no traffic is allowed between e.g., a device in a Multimedia vSpace and a device in a Home Security vSpace. Furthermore, policies may be enforced to prevent unwanted communication when for example an untrusted device is added to a slice containing one or more sensitive devices;
- *Cross-domain resource sharing*, enabling easy discovery of and interaction with third party resources, and securely sharing resources with others. Through simple mechanisms, an authorized user can discover resources of a visited domain and request the establishment of a cross-domain Virtual Space, combining resources of the other domain with some of her own resources, de facto leading to the sharing of devices across authorized users of both domains;
- *Ease-of-use & automation*, allowing intuitive and secure management of all user connected resources and the deployment of task-specific automation processes to support users in different application contexts. Through a simple user interface, a user can see the status of the Virtual Place and manipulate its devices and Virtual Spaces and discover others’. To avoid overloading the user, automation mechanisms allow for dynamic creation and modification of virtual spaces and the associated connectivity.

Section 7 describes a comprehensive user scenario covering most of these aspects.

### 3.2. Virtual Places and Virtual Spaces

To achieve its goals, the Future Spaces solution relies on two key concepts introduced earlier: Virtual Places (vPlaces) and Virtual Spaces (vSpaces): A vPlace is the collection of all resources registered at a network administrative domain, along with all available static properties as well as relevant dynamic states (e.g., connected or disconnected) of those resources. This could include not only all owned devices in the user’s primary home, but also other devices owned by the user at other physical locations (e.g., holiday home, connected car or mobile devices). The vPlace concept illustrates the evolution of CPS towards hybrid environments, where the frontier between the physical and the digital world is more and more blurred, enabling multiple physical domains to be grouped into a single virtual domain. It is important to stress that a vPlace should be interpreted as a managed list of accessible owned resources and their state, regardless of actual connectivity. While a vPlace corresponds to a single domain, it is likely to be multi-user as different users may coexist within the vPlace and have different rights on its resources. In the example of Section 7, two different vPlaces are illustrated, the user’s home vPlace—temporarily extended to her hotel room—and the hotel vPlace, while two users interact with resources (the main user and a visitor) of the home vPlace.

**Figure 1:**
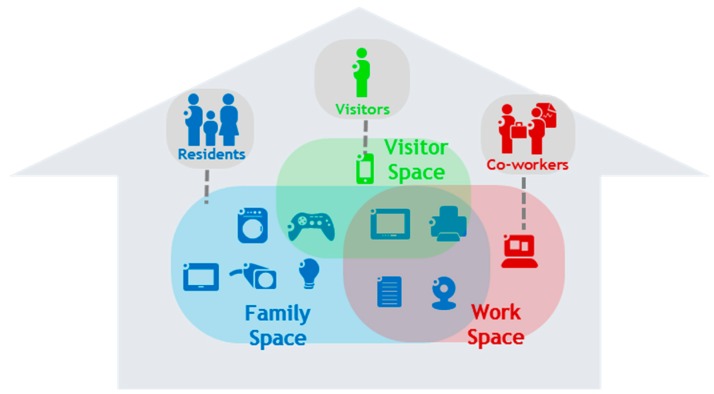
Virtual Spaces regroup and connect resources of a Virtual Place.

To enforce fine-grained control over the connectivity of resources, actual communication in Future Spaces is based on vSpaces. While the vPlace is akin to your virtual home, vSpaces could be seen as different virtual rooms providing secured communication and/or access between selected resources and segregating various activities. For example, in Figure 1 three vSpaces have been defined for different contexts: providing access and connectivity across family devices, across work-related devices and defining a set of devices available to visitors. vSpaces provide isolation by allowing communication between resources belonging to the same vSpace, while blocking all other communication. It is worth noting that a given device can belong to multiple vSpaces at the same time, and that vSpaces can cross vPlace boundaries, i.e., a cross-domain vSpace can be composed of devices from different network administrative domains (homes). In the scenario of Section 7, activity-oriented vSpaces are illustrated: a Media vSpace regrouping multimedia devices from the home (and temporarily from the hotel room) and a Security vSpace for home security devices. Virtual Spaces can be very dynamic, following the addition/removal of devices resulting from their discovery on the network, a change of definition (e.g., by a user-triggered command or automata) or broader context events thanks to automation support (e.g., predicate-based vSpace definition as further detailed in Section 6.3).

Not illustrated through the examples of Figure 1, vSpaces could be augmented through targeted activity awareness. In such cases the role of each device in a group is not fully equivalent. This new notion may help to reduce the number of vSpaces to create in order to limit mental overload on users while increasing security (see Section 5). For example, if a dedicated activity “Printing vSpace” includes a printer and several other resources such as computers and smartphones, then only communication between terminals and the printer should be allowed. In this printing activity, the printer has a central role and thus the inter-terminal communication is not required. This enhanced connectivity model reduces the possibility of security threats through further reduction of attack surfaces if one of the terminal has a data breach. Obviously, it is possible for the users to create one specific “Printing vSpace” per terminal but this would cause too many undesirable user interactions and number of vSpaces to manage. Similarly, communications inside this vSpace could be reduced to only protocols dedicated to printing service and printer discovery. The rest of this article will not develop all these refinements and consider vSpaces at a coarser level such as depicted in Figure 1 for the sake of simplicity.

### 3.3. The Future Spaces Solution

To enforce the vSpaces, the Future Spaces solution enables the seamless creation of a dynamic connectivity layer, implementing a LAN-like connectivity between components of a vSpace by programming virtualized network functions connecting the corresponding physical devices. We call such slices “Software-Defined LANs” (SD-LANs) and each vSpace definition is ultimately embodied as a SD-LAN. To support this type of connectivity, the solution relies on three core functional components:

- a set of Virtualized Network Functions (DHCP, DNS, Internet Gateway and multiple Open vSwitches or OVS [27]), running either inside Future Spaces Gateways (FSGW) or the Future Spaces cloud,
- a SDN Controller (SDN-C) to pilot the virtual switches,
- a MajordHome component in charge of managing the vPlace resources, users and vSpaces, of interacting with the users or automation processes and of turning vSpaces definitions into SDN-C commands.

**Figure 2:**
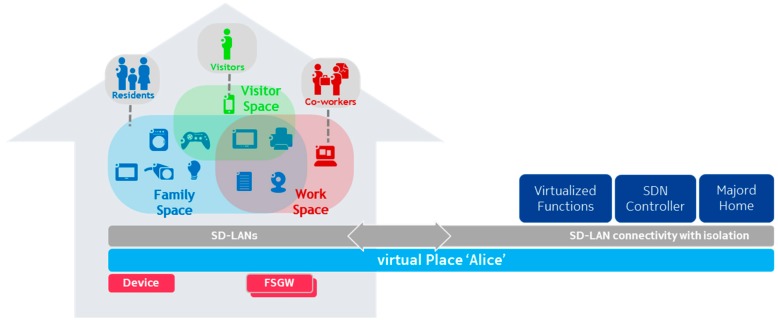
Key functional elements of a vPlace.

Figure 2 presents the simplified architecture for one domain (i.e., a vPlace), which includes here a single physical home.

**Figure 3:**
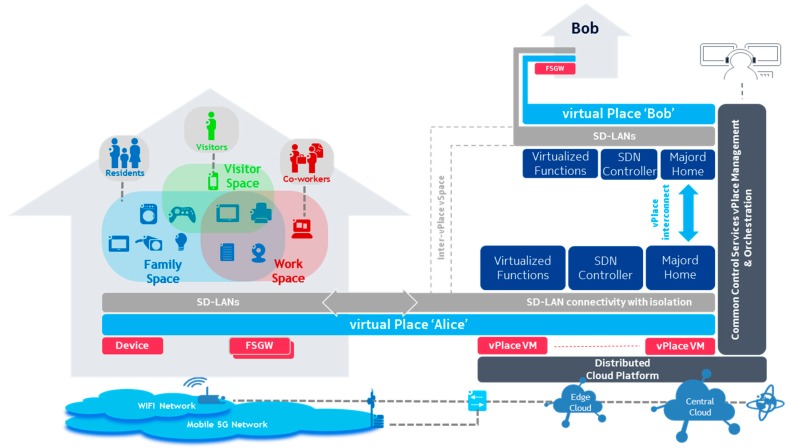
Multiple vPlaces running in the Future Spaces distributed cloud platform.

In our proof of concept prototype, the core functional components are realized as micro-services deployed and orchestrated within a distributed cloud platform (Figure 3). The cloud platform interconnects each FSGW with their corresponding vPlace components, possibly interconnecting multiple vPlaces (here Bob and Alice’s homes). It also provides common control services for vPlace management and orchestration which we describe in Section 4.

All these different software components and supporting infrastructure together realize the Future Spaces solution, for which we implemented a proof of concept. The next section describes the system architecture in more details.

## 4. Future Spaces Proof of Concept System Architecture and Design Choices

Implementing such a complex yet flexible end-to-end solution requires a robust architecture that can automatically scale and adapt itself to the dynamically changing, fine-grained security and performance requirements and constraints. In this section, we discuss the key elements of our software and deployment architecture: the supporting cloud architecture in Section 4.1, the hardware Future Spaces Gateways elements in Section 4.2, the vPlace orchestration and lifecycle management in Section 4.3 and the inter-vPlace tunneling mechanisms in Section 4.4.

### 4.1. Distributed Cloud Architecture

**Figure 4:**
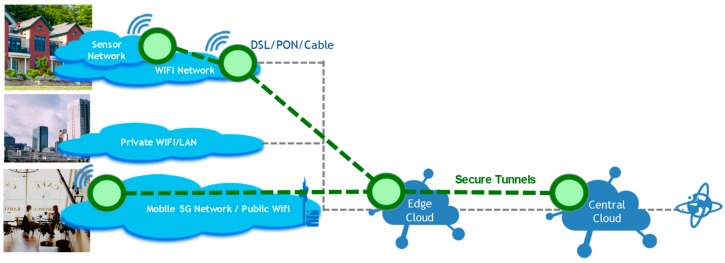
Distributed Future Spaces cloud architecture.

In Figure 4, we present a high-level view of our distributed cloud architecture. Each green node in this figure represents a “Future Spaces instance”, which is a physical or virtual execution environment comprising a common (or thin) Future Spaces management layer/function onto which service components of our system are deployed. These instances are dynamically interconnected using secure tunnels in an ad-hoc secure virtualized network, and can be deployed in a central cloud, edge cloud or even home cloud, depending on use cases and privacy/security/Quality of Service requirements (e.g., based on the devices, services or user’s preferences). 

Some classes of Future Spaces instances provide pure compute and/or storage capabilities whereas others host virtual network functions, or act as a Future Spaces Gateway to interconnect devices (see Section 4.2). Depending on various resource and functional requirements, these instances may be instantiated on a very broad variety of execution environments, ranging from tiny dedicated physical IoT devices to Virtual Machines (VMs) deployed on existing public or private cloud infrastructures—enabling more feature-rich, global and dynamic configuration management use cases.

### 4.2. Future Spaces Gateways

A Future Spaces Gateway (FSGW) is an important class of Future Spaces instance whose main function is to provide secured and managed fine-grained access control via a set of dedicated network interfaces towards the Future Spaces cloud network and its associated/interconnected devices and services. As for any Future Spaces instance, the FSGW can be implemented as a physical or virtual gateway and provides secured access through a number of physical or virtual network interfaces in a device-agnostic manner (i.e., without having to modify or install any specific software onto the devices themselves to achieve this secured access). It implements the fine-grained access control mechanisms (e.g., coming from the SDN-C see Section 5) but also facilitates the onboarding process of the devices onto a vPlace. Furthermore, it comprises services to locally monitor key telemetry data as well as operational events, and to transmit those over the dedicated control plane overlay network via an internal message bus to a central event broker running in the cloud.

**Figure 5:**
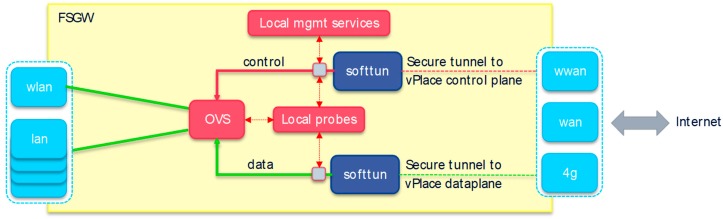
High-level FSGW architecture.

It is important to note that in our current design, each FSGW instance belongs to one specific vPlace, and hence only one local SDN-C and device manager can directly control each individual instance. The overall FSGW architecture is depicted in Figure 5.

On the left, the various local physical or virtual interfaces are depicted, which are used to connect end-user devices and/or services to a particular vPlace. All these local interfaces are under direct control of a virtual switching service that is remotely managed (using OpenFlow commands) by the SDN-C. In our PoC implementation, we used Open vSwitch (OVS) as our virtual switch. As shown in the figure hereabove, the device-related data traffic is independent and isolated from the control traffic, which besides providing obvious security advantages offers additional possibilities as described further.

For this, we set up two separate secure tunnels towards our Future Spaces cloud platform over one or more WAN interfaces. More details on how this process is initiated and bootstrapped, can be found in Section 4.3. In our prototype, we support WAN, WWAN and 4G, and we support that e.g., the WWAN and WLAN interface share the same radio. It is important to state that, unlike in classical VPN solutions, we only need these two tunnels in our architecture to implement all fine-grained cross-location inter-device communication that is required for managing virtual spaces (and inter vPlace connectivity, see Section 4.4). Moreover, it should also be stressed that both tunnels do not necessarily need to connect to the same remote tunnel endpoint. For example, for latency, security or legal reasons, it is perfectly possible to terminate the data traffic tunnel in a closely located edge cloud while letting the control traffic terminate much deeper in the network. Furthermore, our architecture allows to create additional data traffic tunnels to address remote Future Spaces instances directly (e.g., edge cloud instances or other FSGWs), e.g., to avoid network tromboning across locations or to facilitate different QoSes in future work (this has not been implemented in the current PoC realization).

In our proof of concept prototype, we have implemented our own lightweight tunneling mechanism called SoftTUN to securely tunnel vPlace control plane and data plane IPv4 and IPv6 traffic transparently and securely across the Internet or WAN network. This tunneling mechanism supports a number of underlay protocols but in our PoC realization we opted for Secure Websockets, to easily bypass any firewalls and/or proxy servers to connect to our prototype cloud platform.

Apart from these core elements, a number of local management services are deployed directly on a FSGW instance, for example to manage the wireless interfaces, or to reconfigure itself based on remote triggers. We also run and manage various probes, e.g., to directly extract flow information from the virtual switch, or to capture key events such as a device connecting or disconnecting to one of the local interfaces, etc. These events are preprocessed and then forwarded over the Future Spaces control plane management network to the cloud using a message bus (MQTT in our PoC implementation). A local client subscribes to the message bus for particular vPlace events and automatically triggers local scripts accordingly. Additional services include both local and server-based remote WiFi authentication password management for the easy and secure onboarding (see Section 8), as well as transparent support services to enable both local and tunneled breakout Internet access and DNS resolving. Tunneled access and resolving may improve privacy and security in mobile use cases (e.g., MiFi FSGW connected to unsecure (W)WAN interface such as in a hotel or a public hotspot). In our prototype, we have implemented the FSGW architecture on top of the OpenWRT Operating System and successfully deployed it onto a range of physical OpenWRT-compatible physical routers (see Section 7.1).

### 4.3. vPlace Cloud Orchestration and Lifecycle Management

One of our key principles for designing and implementing our Future Spaces cloud platform was to decouple the resource allocation and provisioning from the service management. Specifically, in our prototype, all Future Spaces services and service components are dynamically deployed as lightweight containers on top of a dynamic grid of Future Spaces instances.

To accomplish this, a dual-layer orchestration architecture decouples infrastructure provisioning distinctively from (micro)-service component deployment for efficient and flexible management of all (virtual) infrastructure nodes and service graphs. A top-level orchestration layer is responsible for allocating and provisioning VMs (or possibly also physical resources) in a possibly distributed, heterogeneous and multi-vendor cloud environment. A vPlace Service Orchestrator (VSO) in its turn is then responsible for placing, deploying and configuring all Future Spaces core and application services inside one of these provisioned VMs.

The top-level Future Spaces orchestration layer consists of two key logical components, namely the vPlace Configuration Manager (VCM) and the vPlace Cloud Orchestrator (VCO). The main role of the VCM is to manage all vPlaces and implement top-level “Create, Read, Update, Delete” (CRUD) operations, next to managing vPlace-specific certificates. The main role of the VCO is to allocate and (pre)provision for each vPlace a number of Future Spaces instances (i.e., VMs or FSGWs) for running vPlace services. A key design decision was to deploy all services of a particular vPlace into its own set of vPlace instances (and as such, to first provision a number of VMs for each vPlace), to simplify the lower-layer orchestration and overlay networking but also mainly to strictly isolate services from different vPlaces from each other for security reasons. As such, in our orchestration architecture, each vPlace consists of one or more (lightweight) Future Spaces VM instances. 

For implementing the lower-level vPlace service orchestrator (VSO), we leveraged the Docker ecosystem for packaging, provisioning and deploying all service components for each of the vPlaces individually. Service container orchestration is managed by an existing Docker container orchestration layer such as Swarm.

**Figure 6:**
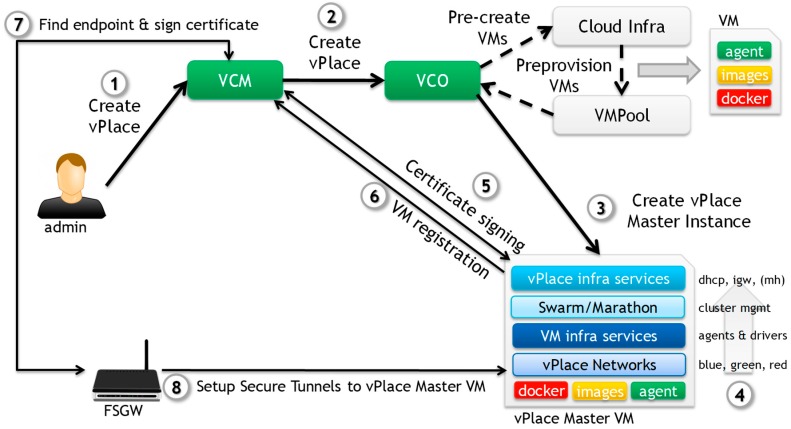
vPlace cloud orchestration and lifecycle management.

Figure 6 depicts the typical high-level life-cycle management of a vPlace. An administrator of the Future Spaces solution first requests the creation of a new vPlace in the VCM User Interface (or using the VCM APIs). As part of this creation, the VCM will contact the VCO to provision an appropriate vPlace VM Instance based on the vPlace configuration parameters and requirements. The VCO will then create a new instance in the appropriate cloud (e.g., central Cloud) and trigger the lower-level orchestration service running in that instance to start provisioning and configuring core services for that vPlace. Note that the VCO typically pre-allocates and pre-provisions a few vPlace instances in our PoC prototype to accelerate the provisioning of new vPlace VM Instances.

The lower-level VSO service orchestrator will first provision the right service image versions from our private Docker registry, and will bootstrap the creation of a fully-functional vPlace Instance, which includes the creation of the various vPlace overlay networks, the core vPlace infrastructure services and drivers, as well as all core management services, such as the SDN-C, MajordHome, etc.

As part of the bootstrapping phase, the VM will generate a VCM-signed certificate and auto-register itself as a new functional vPlace Instance to the VCM, which we call the vPlace Master VM. This certificate is used to set up an encrypted (TLS) tunnel. When a FSGW gets turned on and is being configured, it will register itself to the VCM using vPlace specific credentials and a unique device identifier or request the VCM to generate a new certificate. Next, the FSGW will be instructed about the vPlace VM Instance(s) in the cloud it should connect to and set up its SoftTUN secure tunnels according to the configuration parameters it received from the VCM during registration. Once that is complete, the FSGW is fully operational and devices can be onboarded onto that FSGW and be managed by the services running in the vPlace Instance(s). All the traffic between the FSGW and the vPlace VM Instance(s) is secured. Note that when a vPlace is destroyed, all its vPlace VM Instances are cleaned and appended back into the respective cloud VM pool so that it can be recycled for other vPlaces. All generated server certificates related to that vPlace are also revoked.

### 4.4. Inter-vPlace Connectivity for Cross-Domain Slice Establishment

All Future Spaces instances by default connect only to other instances of the same vPlace. However, we also support connecting devices from multiple vPlaces in the same vSpace. For this, our Future Spaces system supports the dynamic creation and removal of ad-hoc inter-vPlace tunnels to securely cross-connect traffic amongst devices belonging to different vPlaces. These inter-vPlace tunnels are set up and managed transparently by the VSO.

**Figure 7:**
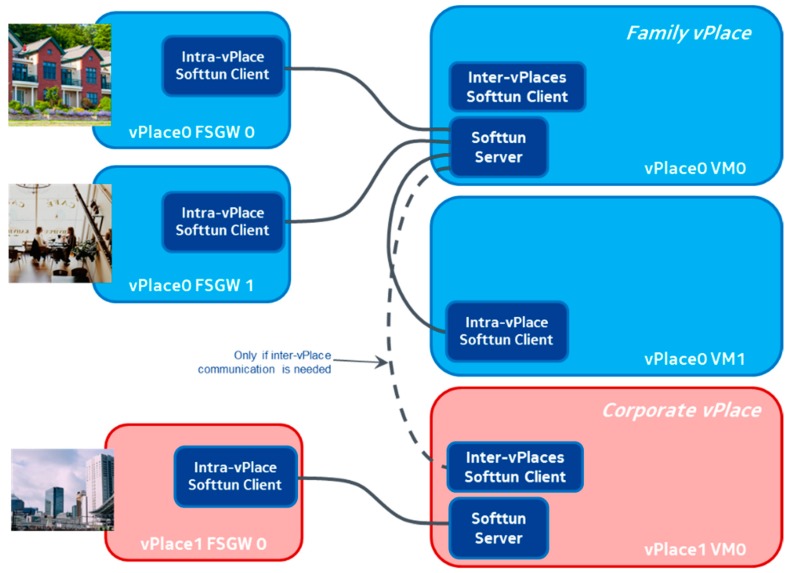
Ad-hoc Inter-vPlace tunneling.

Furthermore, it is important to stress again that only one logical tunnel needs to be set up in between any two vPlaces, though for efficiency and/or security, it may be necessary or beneficial to set up multiple ad-hoc tunnels in between particular Future Spaces instances of different vPlaces. As presented in Figure 7 (dotted-line), the inter-vPlace tunnel is implemented in our prototype as a tunnel initiated from the inter-vPlace SoftTUN client of one vPlace (e.g., corporate vPlace) to the SoftTUN server of the other vPlace (e.g., Family vPlace). Thanks to such a logical tunnel, the two Master OVSes are interconnected together, establishing an inter-vPlace data path. A device connected to a FSGW from a private vPlace could communicate with a device connected to a FSGW from the corporate vPlace via the inter-vPlace tunnel, provided of course that both devices are in one the same cross-domain virtual space, and it is the responsibility of each of the vPlace managers and SDN-Cs that this is properly implemented on each of their Future Spaces networks.

## 5. Software-Defined LAN as the Networking Building Block

### 5.1. SD-LAN Characteristics

As introduced in Section 3, the connectivity of each vSpace is embodied as a SD-LAN. Each SD-LAN implements a LAN-like behavior in terms of data unicast and data multicast/broadcast traffic. This enables full compatibility with all existing home-based network layer 2+ protocols on SD-LANs (e.g., service discovery protocols). However, there are some fundamental differences with the traditional LAN:

- SD-LANs are isolated from each other in terms of traffic, forming a logical partition of the local network at the data plane level. One could say that SD-LANs are equivalent to virtual LANs (VLANs defined in the IEEE 802.1Q standard), but contrary to the VLAN case, a device with a single network interface can be part of multiple SD-LANs at the same time;
- An SD-LAN can span across multiple domains (i.e., local networks of different vPlaces).

Pankratov proposed a “LAN over Internet” solution through the creation of the freeware Hamachi in 2004 [28]. It is a VPN application establishing direct links between personal computers or smartphones, even behind NAT firewalls. This zero-conf product only requires each device to know the identity name of the group they belong to. The software creates a virtual network interface on the devices and a single broadcast domain between all them. But as mentioned in Section 3.1 we strongly advocate for no modification of the devices (in particular through software installation) as we would like to also address consumer products such as printers, thermal sensors, etc.

From the perspective of the devices, the SD-LAN is completely transparent. Devices continue to behave as if they were connected to a single traditional home LAN. In practice, their network traffic is potentially handled and forwarded by multiple SD-LANs depending on the number of vSpaces they belong to. It is the responsibility of the SDN-C to implement different forwarding graphs in order to segregate traffics between SD-LANs on the same physical infrastructure (network underlay).

Our SD-LAN concept derives from the zero-trust model [29], i.e., that the network inside the domain perimeter (the home in the context of this article), cannot be trusted due to the growing number of resources plugged into that network offering a large range of attack surfaces. The SD-LAN concept relies on Identity Driven Networking and Application Driven Networking approaches to not only provide access to granted data (based on source and destination devices, locations, time and data itself) but also to incorporate them on provisioned SD-LANs which thinly segment that home network. Thus, through the creation of these micro-perimeters, we drastically reduce attack surfaces, despite SD-LANs covering multiple domains in some usage scenarios. To quote Kindervag, SD-LAN offers a solution such that “the packet only gets access to approved resources at the approved time”. Moreover, the data traffic could be analyzed per SD-LAN to check that it is conforming to the forecast activity (e.g., a SD-LAN dedicated to printing between a set of computers and a printer should only allow computer to printer traffic).

### 5.2. SD-LAN Underlay Architecture

To enforce SD-LAN configurations, we rely on a distributed architecture of custom Software-Defined Network Controllers (SDN-C) consisting of one SDN-C per domain (vPlace). The SDN-C north-bound interface towards the MajordHome allows to inform the latter of device information (e.g., MAC address, location, etc.) and conversely to retrieve different policy rules to enforce on the data plane. As we can see later in Section 6, these policy rules received from the MajordHome’s vSpace manager derive from different users’ intents or context changes.

**Figure 8:**
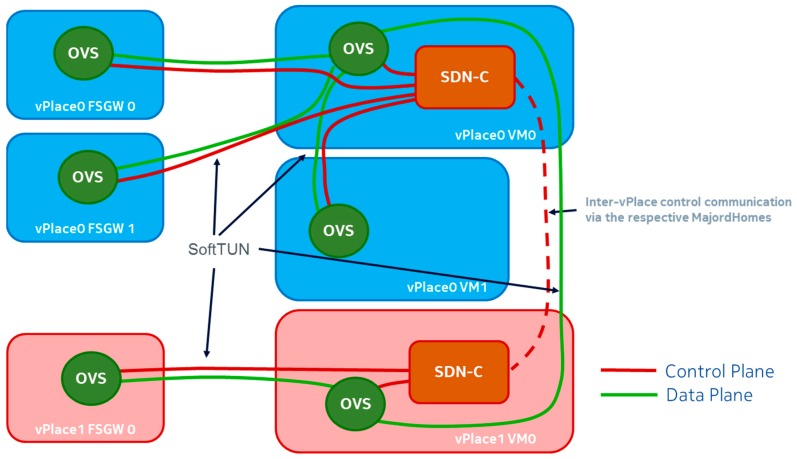
High-level SDN architecture.

Each SDN-C is responsible for controlling network equipment located in different Future Spaces instances (e.g., FSGW or VMs) belonging to the associated vPlace. In our Proof of Concept (PoC) prototype, we rely on Open Flow SDN switches (i.e., OVSes). Note that other implementation choices are possible to realize the SD-LAN concept. To control network equipment, we rely on the dedicated (i.e., out-of-band) virtualized control infrastructure described in Section 4 (red lines in Figure 8). This allows for the exchange of OpenFlow control messages between controlled entities (i.e., OVSes) and the SDN-C. When an SD-LAN spans across multiple vPlaces, SDN-Cs across those vPlaces collaborate with each other, via their respective MajordHomes and the ad-hoc inter-vPlace tunnel link, to implement the entire SD-LAN. In this case, each involved SDN-C is responsible for enforcing the SD-LAN partition within its vPlace boundaries as flow entries in its OVSes.

In our PoC prototype, at the data plane level, OVSes located within different instances of a given vPlace are connected to the OVS of the vPlace Master VM via dedicated (i.e., data plane only) secure tunnels. Ad-hoc inter-vPlace tunnels can also be setup to interconnect the vPlace Master OVSes when inter-vPlace data traffic is required (e.g., cross-domain vSpace). As already mentioned in Section 4.2, our current PoC does not implement tunnels between FSGWs, but this can be realized as a future step as the SDN-C implementation for this PoC prototype is independent of the network topology of OVSes, it only needs to know about the nature of its different data plane interfaces. The latter can be of three different types: either they are connected to another OVS of the same vPlace (intra-vPlace connection), or they are connected to other vPlaces (inter-vPlace connection), or they are connected to devices. Note that these devices can be end-user resources or Network Functions (DHCP, Internet gateway, data traffic analysis …). As external tunnels towards other vPlaces are setup according to operational needs, the SDN-C is aware of the destinations of the corresponding interfaces on OVSes. Note that the current SDN-C prototype implementation used in the PoC has limitations relative to switching loops—we discuss those further in Section 8.

### 5.3. Operation Description

To enforce fine-grained network control, the modification of a vSpace can trigger a new configuration of the underlying networks. Such modification can result from a number of events:

- The MajordHome can send configuration orders to include/remove a device in/from the vSpace. The reason for such change can be the modification of the vSpace definition through user interaction, or the change of reachability of a device in this vSpace, or more generally because of an automated reevaluation of the vSpace content (see discussion on automation in Section 6.3). For example, a device discovered as compliant with a given property (e.g., “DLNA-compliancy”) would be automatically added to a vSpace regrouping all devices matching this property;
- A vPlace can be extended to a new physical location, through the addition of a new Future Spaces instance (FSGW);
- A vSpace can be extended to another existing vPlace, e.g., to include a device from a third party or to follow the mobility of a device in a visited vPlace.

Other network configuration changes can result from events or policies not necessarily managed by (or even visible to) the user. An example is the integration of Virtual Network Functions (VNFs) such as Firewalls, DHCP services or access to the MajordHome, necessary to the operation of the vPlace. Another example is the application of general policies such as the default provisioning of the Internet access for selected devices, where each concerned device may be allowed communication with the Internet Gateway, but not necessarily with other devices.

Finally, switches have to be configured to handle classical device mobility (intra-vPlace) but also roaming across domains (inter-vPlace) in order to maintain SD-LANs forwarding.

The SDN-C prototype has been implemented to support different network infrastructure events such as the dis/appearance of controlled network equipment (e.g., upon startup or shutdown of a FSGW) or network equipment mobility (e.g., MiFi mobility). Each SDN-C, operating under a single vPlace, builds the forwarding graphs realizing the SD-LANs corresponding to the different network partitions (or network segments in security vocabulary). This involves synthesizing rules for unicast and multicast/broadcast traffic that are enforced atop the shared vPlace network infrastructure. As mentioned in Section 5.1, Future Spaces relies on a zero-trust network model, meaning that network connectivity is created and updated according to real and dynamic needs. Unicast traffic forwarding relies on dynamic whitelisting enforced on network equipment, based on source-destination pairs. In some scenario, application services can also be segregated though analysis of the data traffic such as filtering on transport layer protocol packet fields. One of the main problems when trying to realize SD-LANs is supporting end-to-end broadcast/multicast traffic across a hierarchy of OVSes, especially since devices can belong to several SD-LANs simultaneously. In this case, for each broadcast frame, the destinations are the devices participating in a SD-LAN containing the source. The solution must therefore ensure that only one copy of the frame is sent to each destination, regardless of the number of shared SD-LANs. For each source device, a logical broadcast tree is computed allowing to define destinations which are allowed to receive its broadcast/multicast traffic. Obviously different implementation choices are possible to find network performance tradeoff (latency, flooding …)—our detailed solution and related discussions can be found in [30]. Similarly, traditional split horizon principle has to be applied in the context of inter-vPlace connections. Finally, the SDN-C has to adapt flow entries to cope with device mobility across networks (intra- and inter-vPlace).

In addition to the software-based automation of a vPlace’s network configuration, each SDN-C is responsible for keeping an up-to-date inventory of the reachability of devices (e.g., though MAC learning). Each SDN-C detects the presence and mobility of devices on the network infrastructure and reports the device’s (dis)connection to the MajordHome.

### 5.4. SDN-C Architecture Solution

**Figure 9:**
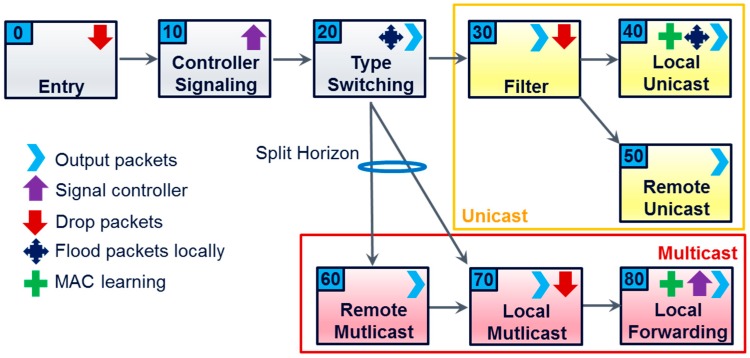
Flow tables for the OVS.

We explained in the sub-sections above that the SDN-C realizes a synthesis of all SD-LANs and configures the OVSes it is responsible for accordingly. The PoC prototype relies on the OpenFlow protocol from release 1.3 onward as it requires multi-tables feature support. As described in [28], and showed in Figure 9, each of the following tables in the OVS plays a specific functional role:

1. Table 0 aims at filtering miss-formatted frames;
2. Table 10 informs the controller of newly discovered devices (includes MAC learning);
3. Table 20 differentiates multicast/broadcast from unicast traffic, and adds different tags to LAN-originated and WAN-originated traffic flows;
4. Table 30 tests pair of source and destination addresses to determine if a unicast frame is to be dropped or forwarded (finer grain filtering is possible to isolate application services);
5. Table 40 is updated through MAC learning and used to forward unicast frames within the local network;
6. Table 50 sends unicast frames to the legitimate remote networks (vPlaces);
7. Table 60 sends multicast frames to the legitimate remote networks (vPlaces);
8. Table 70 sends multicast packets to the legitimate destinations in the local network;
9. Table 80 is updated through MAC learning to forward multicast frames according to a new LAN multicast tree.

According to the events listed in the previous section, the OpenFlow switches are configured by adding or removing flow entries in the above flow tables with different priorities. Reference [30] describes the SDN-C procedures to react to a live network and the algorithm which allows for implementing an end-to-end multicast tree for each registered device. When an incoming Ethernet frame enters in the interface of a switch (named “port” in OpenFlow), the frame is handled as a packet (in OpenFlow terminology) by the flow table 0. Tables apply instructions according to the matching of this packet to a flow entry during the lookup process. The principal instructions that we are using are: forward the packet to another table for further processing (see possible arrows in Figure 9), drop the packet, forward the packet to the SDN-C through the OpenFlow control channel—principally when the source MAC address is unknown as per the MAC learning process (table 10)—or forward the packet to one or several output ports of the switch. As already mentioned, when the SDN-C receives a packet from an OVS, it reacts either through new network configuration if the source MAC address is already known on its side or by transmitting the information through its north interface to the MajordHome.

The number of flow entries to insert inside the flow tables for OVSes of a given vPlace is dependent on the number N1 of devices belonging to that vPlace, the number N2 of devices from other vPlaces participating in vSpaces with some of them, and the number N3 of special devices such as VNFs (e.g., DHCP server, MajordHome and Internet gateway). If we assume that there are N = N1 + N2 + N3 devices to consider, then per OVS there are a maximum of 5N flow entries distributed in tables 10, 40, 50, 60, 70 and 80 and N × (N1 + N3) entries in table 30. If we consider that some vSpaces include many devices from other vPlaces then N2 can increase drastically. In a home scenario we assume that this value will not be at a level which represents an issue for the performance of the forwarding. Such problematic consideration must be evaluated in other application contexts such as enterprise or smart city settings where we can imagine scenarios with millions of devices. But in such contexts, other choices of implementation are possible to reduce the impact of the number of devices on the network equipment.

## 6. Automation and User Support in Future Spaces

By abstracting the network complexity and easing its management with the vSpace and vPlace concepts, Future Spaces offers a secure playground for home IoT environments and a promising framework for CPS related scenarios. With that approach in mind, we implemented user-friendly configuration management tools as well as easily programmable mechanisms for specifying behavior automation to naturally and securely manage IoT devices from Future Spaces-enabled homes.

The component centralizing the user interactions and supporting the automation features is named the MajordHome [31]. It is essentially an OSGI-based backend server in charge of making the link between the vPlace constituents (i.e., all owned resources) and vSpaces on behalf of users and is realized as a set of components exposing APIs. Through these APIs, the MajordHome is receiving requests for configuration changes. It also receives notifications of changes in network states advertised by the SDN-C and other network functions of the vPlace (such as DHCP/DNS information). Based on these updates, automation processes of the MajordHome insure the consistency between the user intention and the internal system states, before instructing the SDN-C of required connectivity changes.

### 6.1. Handling of User Interactions and Customizations

Permission management is the cornerstone of the MajordHome, as it directly controls which resources can communicate to what other resources and how. Ownership and access control rules, applied to the three main entities of the MajordHome (User/Roles, vSpaces and Devices) are stored within the vPlace and define most of the mechanisms: vSpace membership, connectivity, discovery, etc. Beyond the fact that Future Spaces design is itself tightly coupled to this access control model, the very nature of IoT devices and their environments (in our case the home) require a strict handling of security and privacy constraints.

The management of a vPlace is achieved by a User whose actions are defined by Roles. Depending on the granted permissions, that user (being e.g., a simple user, manager, owner or visitor) will be able to access different functions through interfaces. To leverage the Future Spaces capabilities across most scenarios, three different types of access-controlled interfaces can be used: (1) an administration user interface (Web and mobile), (2) an HTTP REST API, and (3) interfaces provided by the OSGI framework.

#### 6.1.1. The Administration User Interfaces

**Figure 10:**
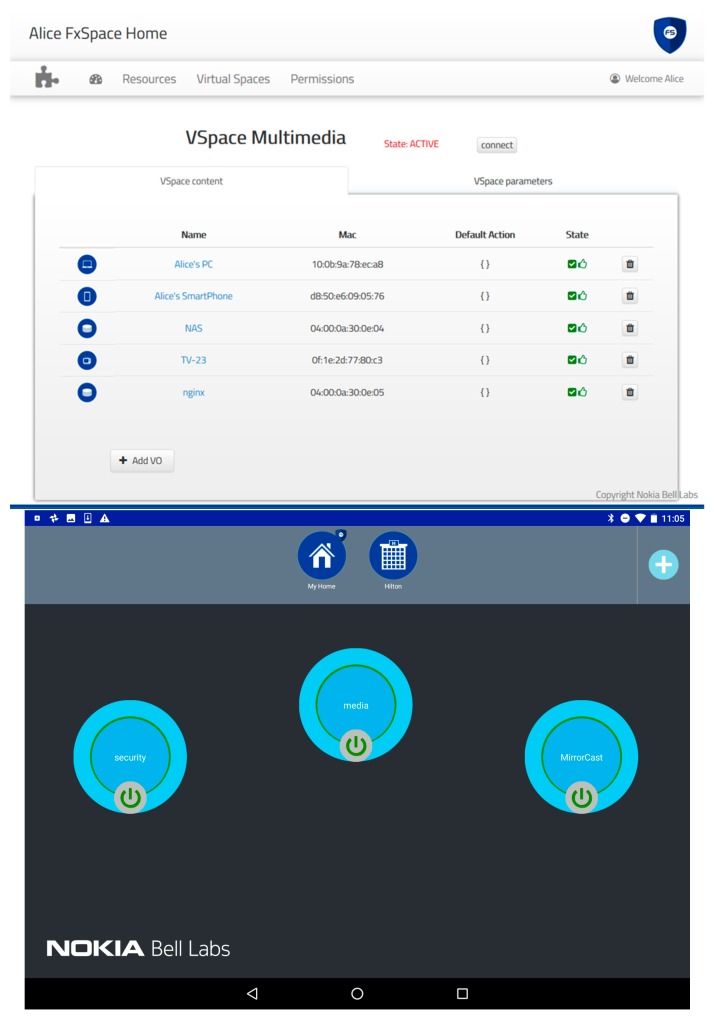
Screenshot of the MajordHome web interface showing the composition of a “Multimedia vSpace” (**top**) and the Android UI (**bottom**).

The administration application is a simple web interface (Figure 10) that enables a user to manage her vPlace. It is automatically available when a vPlace is created and allows authorized users to perform most administration tasks over her Home environment. It could be accessed through a specific URL (provided along with credentials) or directly when connecting to the FSGW (captive portal). A non-admin interface could also be served the same way, offering basic services and information to visiting devices/users.

A secondary administration application is also available on Android devices (Figure 10 Bottom). It provides a handy companion for the vPlace administrator thanks to notifications (e.g., on newly detected devices) and the ability to add new appliances through proximity communication technologies (such as NFC) or by flashing a Quick Response code (QRcode).

#### 6.1.2. The HTTP REST API

The previously presented administration applications are basic tools that allow immediate use and management of the vPlaces by the users. However, those are mere examples of clients leveraging the Future Spaces REST API. This REST interface was designed to allow third-party developers to implement various applications over the Future Spaces framework: e.g., managing connectivity between devices over multiple vPlaces, automatically registering new devices, etc. The possibilities are endless as long as the client has the necessary permissions.

#### 6.1.3. The OSGI Framework

Finally, at the lowest level of abstraction (in terms of programming stack), MajordHome core mechanisms are also exposed in a modular way, thanks to the OSGI framework. This enables allowed third-party developers to easily implement plugins (aka “bundles”) that could be integrated into the MajordHome itself, addressing CPU-consuming and time-sensitive requirements. This framework is also used internally to provide more flexibility, to dynamically update and adapt to the environment requirements and selected configuration. Additionally, Future Spaces comes with a bundle repository which can be seen as a kind of “app market” for vPlace services that could be selected and installed by the user to suit her/his needs.

### 6.2. Architecture and Data Model

To perform its duties, the MajordHome is manipulating a representation of the physical world (a Data Model), which mainly consists of the following entities:

- VirtualObjects: to model the devices connected to the network, and carry information concerning their capabilities and their attributes,
- VirtualSpaces: to model the SD-LANs and hold the composition of these SD-LANs in terms of devices,
- Behaviors: to model simple automata,
- Users and their Roles: to model the users and control their permissions in interacting with the MajordHome,
- Sharing policies: to model which constraints users want to enforce when sharing their devices (e.g., making sure that the door lock securing system of their house cannot be shared with untrusted devices).

**Figure 11:**
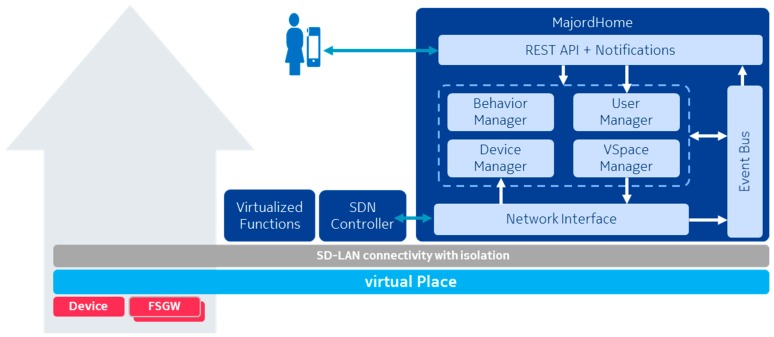
Architecture of the MajordHome showing interfaces with other components of the Future Spaces solution.

The first four elements are independent entities which are managed by dedicated functional blocks of the MajordHome realized as OSGI bundles, while the sharing policies are defined in the scope of either a VirtualSpace or a VirtualObject. These four first elements have both a Data Model representation and a software agent to provide the proper interaction with the MajordHome functions. Figure 11 depicts functional blocks of a MajordHome in relations with the other elements.

The core blocks are the four managers (VirtualObjects, VirtualSpaces, Users and Behaviors), which are managing for their respective Data Model entities their persistency, their modifications and the references to the dedicated software agents (each agent in charge of keeping up-to-date state information and performing interactions with other entities). These four managers and the agents of their entities interact with each other either directly or through events (communicated over the OSGI event bus).

The REST API and Notifications functional block is gathering interfaces with the end-user (see previous section). Upon a request (from user client or automation agent), this block starts by checking the permissions of the requesting entity (call to User Manager). If granted, the request is passed to the appropriate manager (or agent), which response is formatted by the block. This block also includes support for WebSocket communication to inform the user clients and agents of modifications in the state of the vPlace. Finally, this block is also responsible for sending specific notifications to targeted users, using external messaging mechanisms.

The final functional block is the Network Interface which is in close relation with the SDN-C. First the SDN-C notifies of network events (e.g., connection status of devices or FSGW), which is then passed to the Device Manager and the VirtualObject agents. Second the network interface instructs the SDN-C of the connectivity to be achieved in between devices. These instructions are derived from the VirtualSpace agents (each VirtualSpace agent is in charge of determining which devices should be connected via its corresponding SD-LAN).

### 6.3. Automation for Users

**Figure 12:**
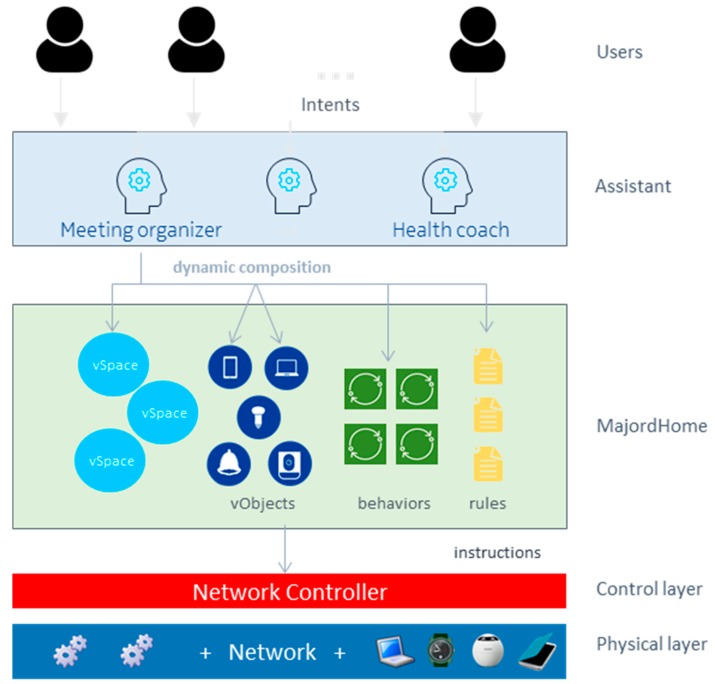
Layered composition of the Future Spaces solution, from user-intents to physical network configuration.

In Future Spaces, automation is realized through a number of mechanisms, within and above the MajordHome component as depicted in Figure 12. The different automation mechanisms are, listed in order of increasing complexity:

- Predicates in VirtualSpace agents: the composition of a VirtualSpace is determined by a Predicate (Boolean function). Every device for which the VirtualObject matches the predicate of a given VirtualSpace will be part of the corresponding SD-LAN. e.g., a vSpace can be composed out of a defined list of devices, or e.g., be automatically composed of every device matching a given characteristic (e.g., being multimedia capable), as soon as they connect to the network.
- Sharing Policies engine: every VirtualSpaces and VirtualObject agents contains such engine to ensure that their sharing policies are respected. Such policy typically expresses context in which their corresponding host should be used (e.g., a policy on a device may state that it cannot take part in a vSpace including devices not belonging to its owner). More details on sharing policies can be found in [32].
- Behaviors: MajordHome supports event-driven automated behaviors, which essentially are callback functions that are automatically triggered by specific events and then perform some modifications on the system (e.g., perform a given task to enrich the information contained in a VirtualObject when a device connects for the first time). These behaviors can be started, stopped and customized by the user. MajordHome plugins coming as OSGi bundles can augment the catalog of behaviors (see Section 6.1).
- Assistants: Third-party assistants handle focused intents of users, and support the automation required to handle these intents and translate those in a set of requests to the MajordHome REST API (limited to the scope of the permissions granted to the assistant). Assistants can be realized as software components external to the MajordHome, allowing to support more complex logic than Behaviors or even be hosted outside of the Future Spaces platform.

Users of a given Future Spaces instance can therefore customize at will their smart environment by mixing the possibilities of adding high-level easy-to-use third-party assistants and customizing their MajordHome instance (with additional bundles and the customization of behaviors, using vSpaces with tailored Predicates, and setting Sharing Policies on their vSpaces and resources)—without having to manually do all the low-level complex network configuration management. 

## 7. Proof of Concept Setup and Functional Use Case 

### 7.1. Proof of Concept Setup

Our proof of concept setup comprises a small custom OpenStack Cloud environment located at our premises, and multiple simulated home environments comprising several devices connected to local FSGWs in various fixed locations. Mobile FSGWs can also connect to the Future Spaces cloud, allowing to connect devices from any location with Internet connectivity. We currently support different models of existing physical gateways, such as a Mobile WiFi router (e.g., TL-MR3040, TP-link, Shenzhen, China) as well as more traditional home or enterprise routers (e.g., TP-link Archer C7 and WR710n (TP-link, Shenzhen, China)). User actions are performed using our Android UI running on a tablet or smartphone. During our PoC validation, we mainly focused on the functional aspects of the end-to-end seamless interconnectivity and isolation capabilities of our system.

The main scenario involves an end-user travelling for business-purpose and arriving at her hotel. She carries with her a mobile FSGW (i.e., Mobile WiFi), which allows her to remain in contact with her smart home and attach securely all devices she is travelling with to her vPlace. The mobile gateway is connected to the hotel WiFi network to access the Internet, and securely connects back to the Future Spaces Cloud. In addition, the user will be able to discover new devices exposed by the hotel room vPlace, and to invite a visitor to join the system and securely interact with devices he has selected, illustrating the multi-user capabilities.

Our PoC setup currently has well over a dozen permanent vPlaces (and corresponding VMs) up-and-running across three different VCO orchestration entities running in in two local clouds at different locations in Europe, about twice the amount of active physical FSGWs connecting to these vPlaces on three continents, and over 100 unique registered devices. Aside from this, we also deployed many more temporary vPlaces for specific internal and external demos as well as smaller functional tests. Apart from a few permanent live setups at various locations (home, office, etc.), we also used our PoC system for numerous live demos, including one live demo where external people from Asia were able to cast a video from their smart phone onto a Chromecast or DNLA-capable device in North America (across one of our clouds in Europe), as if that casting device was in the same room without any inconvenience that would translate a perception of distance. Using our Android UI client, we could very easily and dynamically give these visiting devices access to these casting devices, or revoke their access, by simply dragging-and-dropping their devices in or out of the corresponding vSpace and let them briefly interact with the remote device as if it was their own device running in their own local (home) network. In the following, we describe the main functionalities of the Android UI, before depicting the various steps of the scenario showcasing the properties of the solution and its easiness of use.

### 7.2. Overview of the Future Spaces UI

**Figure 13:**
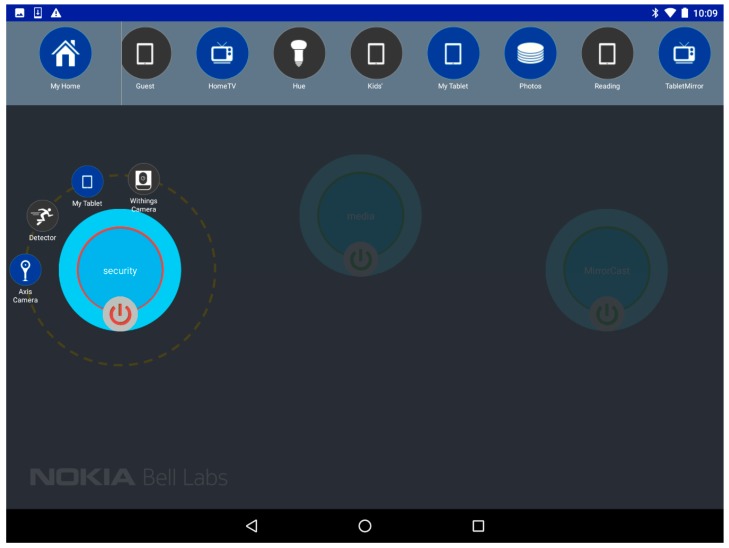
User Interface of the Future Spaces application on an Android tablet.

To provide an intuitive interface for the user to stay in control of her connected environment, the Android UI provides the important information to the user, and allows simple drag-and-drop-based interactions to create and update vSpaces. Figure 13 shows two screenshots of the UI. On the top of the screen, we can see the different vPlaces (collapsed view on the left) and the devices (expanded view on the right) the user is granted access to. The user can scroll right/left through all available devices of a vPlace and is informed of their status (Blue = connected; Grey = disconnected). Mechanisms to add other vPlaces to the list of vPlaces accessible to the user can be triggered by the “+” icon.

The user can select a device icon, drag and drop it either on an existing vSpace (represented by the big bubbles on the main section of the screen), or on an empty spot which will automatically trigger the creation of a new vSpace. Figure 13 shows two possible representations of vSpaces: a collapsed view with only the vSpace name (e.g., the “security” vSpace on Figure 13 right) and a button to connect or disconnect it (which instantaneously connects—green circle—disconnects—red circle—all devices of the vSpace by enforcing/disabling the corresponding SD-LAN), or an expanded view where the user can see all devices included in the vSpace. A simple click on the vSpace bubble expands/collapses between these two views.

### 7.3. Step 1: Stay Connected to Your Smart Home on the Move

The user connects her tablet to her mobile gateway and launches its Future Spaces client application. She then sees all her devices, both back at home connected through her fixed FSGW there and the devices she may have brought and connected to the MiFi FSGW. She can use any application she would use normally at home to benefit from her connected devices and services.

### 7.4. Step 2: Allow/Block Communication of a Group of Devices in a Single Click

As a first action, the user can try to visualize the feed of her connected camera at home, which relies on local communication between the tablet and the camera. At first the associated vSpace (“Security”) is temporally disconnected (red lining in Figure 13) so it is not possible for the tablet to connect to the camera. Simply pressing the on/off button of the vSpace representation in the UI allows to activate the communication between all devices of this vSpace, resulting in the SDN-C to setup the necessary forwarding rules in the OVSes of the vPlace. As a result, the user can effectively monitor what is happening in her home by directing her device browser to the video-serving embedded web browser of the Camera device.

### 7.5. Step 3: Interact with Other Smart Environments and Discover & Use New Devices

**Figure 14:**
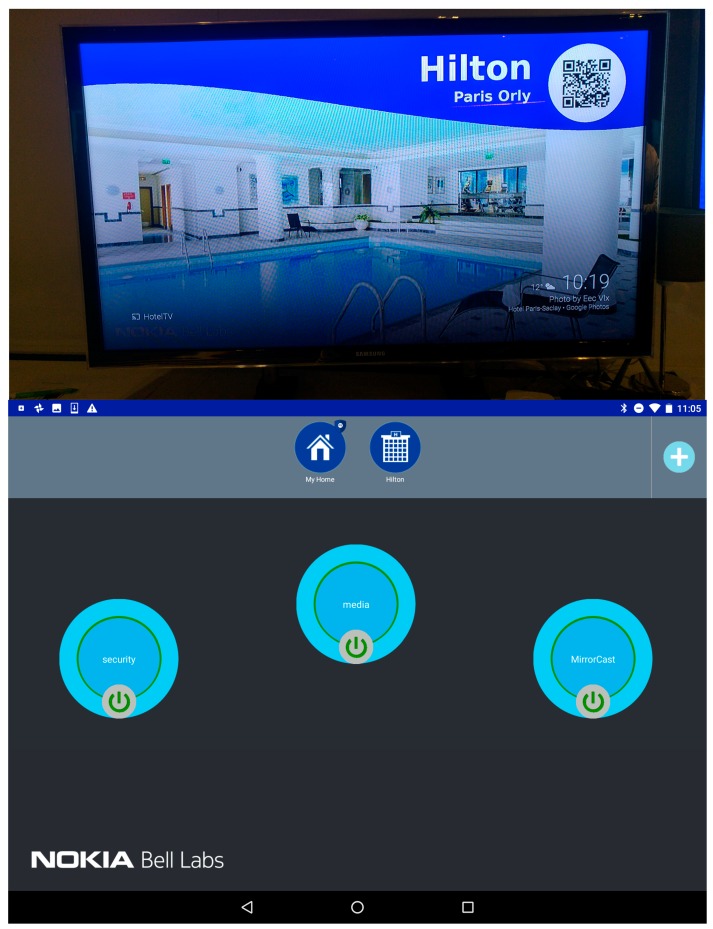
Scan of the QR code on the hotel TV to discover the hotel room vPlace.

The promise of the Internet of Things is also to allow the user to interact with her environment and third-party devices and services (e.g., from the hotel in our case). She first needs to discover what the hotel is willing to expose, e.g., by scanning the QR code on her hotel TV screen, which reveals a new vPlace in the Future Spaces UI (Figure 14).

From the available devices in her room served by the added vPlace, the user decides to connect the hotel TV to her personal “Media” vSpace, by dragging the TV icon from the Hotel vPlace to the Media vSpace bubble. This automatically programs the corresponding network nodes across the two vPlaces, and the hotel TV can now be discovered by home DLNA devices of the Media vSpace through the multicast SSDP protocol. As a result, the user can cast content directly from her personal storage at home or from her personal cloud storage onto the hotel TV although both are attached to different domains.

### 7.6. Step 4: Easily Provide a Secured Access to Visitors and Be Warned of New Events 

**Figure 15:**
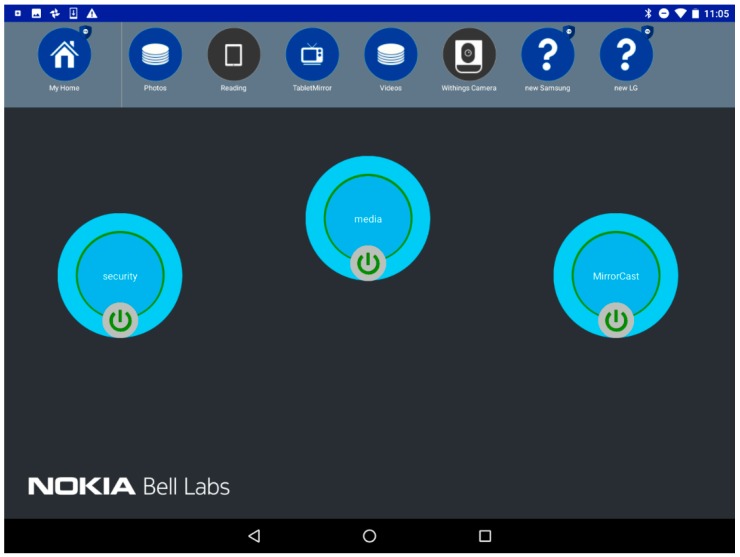
Warning of an event on the home vPlace and discovery of newly connected devices.

Apart from interacting with third-party devices and services, the user can also easily give a visitor smartphone temporary access to her vPlace, and control which devices the smartphone can connect to. For this, the visitor first connects his smartphone to the (mobile) FSGW of the user. Once the new device is on-boarded, a shield appears on the corresponding vPlace in the Future Spaces user interface to indicate that a new event occurred, and by looking at the devices in the vPlace, the user discovers a new unknown device, also tagged with a shield (Figure 15).

Until it is assigned to a vSpace, the visitor smartphone is only connected to the local mobile FSGW, it has no connectivity to the user’s devices at home or even no connectivity to the Internet, demonstrating a strong isolation policy configured in the system. Dragging the smartphone to the Media vSpace will enable LAN-like communication between this new device and other devices of the vSpace, e.g., the visitor can start its YouTube application to discover the remote home TV (relying on mDNS protocol) and start a video there.

### 7.7. Step 5: Safeguards on Devices and Virtual Spaces Sharing 

**Figure 16:**
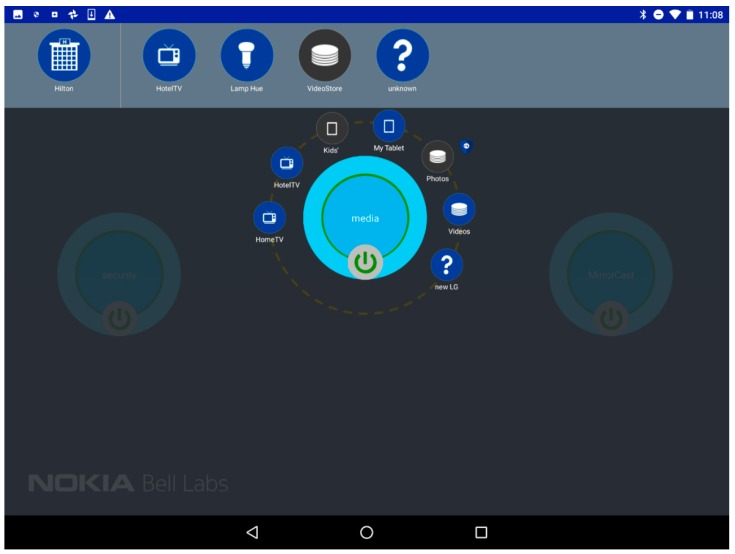
Automatic exclusion of the private photo disk in presence of an unknown device.

Through the policy mechanisms depicted in Section 6.3, the solution can enforce more security in the way the user can share her devices with others through vSpaces. In the Media vSpace, a policy was predefined so that the Network Attached Storage (NAS) with the family photos has to be disconnected if an unknown device is added to the Media vSpace in the previous step. This is exactly what happened when the visitor smartphone was added in the previous step. The Future Spaces UI reflects the security policy enforcement by displaying a shield on the Media vSpaces, and upon expansion of the vSpace the user notices that the new device was indeed included, but also that the Family Photo disk has been temporally excluded (Figure 16). This is the result of the evaluation of the policy attached to the disk, triggered by the new event of introducing the visitor smartphone in the Media vSpace. Removing the smartphone (by dragging it from the vSpace to the trash bin at the bottom right corner of the UI), the policy is re-evaluated, and the disk is automatically reconnected.

## 8. Discussion

In this section, we discuss limitations and open issues of our proof of concept, reflecting back on the key aspects detailed in Section 3.1.

### 8.1. SD-LANs as a Solution for Fine-Grained, Context-Aware Access Control

The cornerstone of the Future Spaces solutions is the Software-Defined LAN concept and realization. While it has shown its applicability and stability in different scenarios, some aspects are worth discussing here. 

First, the SD-LAN concept has been designed with the assumption that it should work for the least common denominator among IP-capable devices, i.e., devices supporting a single IP network interface that is not in itself “sliceable” (it cannot be partitioned into different logical entities, each of them potentially connected to a different network slice). In the upcoming 5G standards however, devices are envisioned to be sliceable and be connected to up to 8 network slices concurrently. This requires the modification of existing device stacks to make them compliant to new 5G standards, which goes against our original working assumptions. 

Second, in this paper we described our current approach which applies only to IP/Ethernet-capable devices; integrating other networking technologies such as BLE or Zigbee is part of our current work. 

Third, as hinted in Section 5.2, the current SDN-C prototype implementation has two limitations relative to switching loops: first, the intra-vPlace underlay network topology should not include loops; second, multiple inter-vPlace tunnels must not be setup toward another vPlace (only one per external vPlace). While it is not a blocking issue, it could be interesting to support network switching loops to enable mapping traffic on different paths. This would enable for example to setup inter-FSGW shortcuts in intra-vPlace multi-location scenarios (e.g., to reduce latency). Other scenarios could be envisioned in inter-vPlace context or relatively to other QoS parameters such as bandwidth.

Finally, our solution is designed targeting an IPv6 environment. However, as current application support of IPv6 is not always correct, our proof of concept is implemented with IPv4 addressing. For this, the PoC prototype manages all vPlaces so that they all are part of the same IPv4 subnet range, enabling cross-domain connectivity at IP layer. Indeed, simple Network Address Translation is not applicable since several widely used LAN application and discovery protocols transmit IP addresses in their payload. Using NAT would require traffic content translation for every type of application communication and would be anyway jeopardized by the increasing amount of encryption of such traffic, while the usage of a logically centralized Future Spaces infrastructure allows to address this aspect by controlling the addressing scheme.

### 8.2. Cross-Domain Resource Sharing via Virtual Spaces

A key differentiating feature of our approach is precisely the cross-domain connectivity allowing resource sharing across homes. While our prototype provides some first proof points, open issues remain for a more general solution. A first item is on how to uniquely identify a device across administrative domains. We relied in our controlled PoC on standard 802.3 MAC addresses assuming their uniqueness, while MAC address spoofing and virtualized environments with virtual MAC addresses should be taken into considerations in future work. Similar questions arise for user identification across domains, as we relied on our logically centralized cloud platform to manage user identities—in a distributed pattern (e.g., multiple ISP with their own identity schemas), another solution should be envisioned. Additionally, in such a context of a Future Spaces solution deployed by multiple ISPs, it would be of interest to also address end-to-end inter-domain QoS to ensure the quality of experience and further enhance security of the realized resource sharing.

In a broader perspective, resource sharing scenarios pose issues similar to those of social networks (such as “with which user do I authorize the sharing on this resource?”) or of file system rights management and inheritance (e.g., determining which user can see a given resource in the MajordHome, which user can read information about this resource, which user can insert this specific resource in its vSpace creation). We took a naive approach in our current implementation of multi-user vSpaces, by providing all contributing users with equal rights and roles. In more complex scenarios, dissymmetrical roles could be envisioned, ruled by contracts for vSpaces, an area which may require inter-disciplinary work with human and social science.

### 8.3. Automatic Security & Privacy Protection

Thanks to the network isolation provided by SD-LANs and the use of automation policies in Virtual Spaces content, our approach allows to greatly improve the security of connected resources and home networks by drastically reducing the attack surface, while providing more control to users over their data privacy by controlling which resources connects where. However, security automation and keeping users in control can be challenging to realize jointly. For instance, collaboration scenarios where multiple users with different objectives and security constraints use vSpaces to interconnect their resources raise some reconciliation (not to mention usability) challenges, for which strategies may be very scenario dependent (e.g., assessing and giving priority to the actor that takes the most risk—a challenge in itself).

Our current approach relies on automatic actions based on the predefined policy rules considering a limited number of events (i.e., a device being added to a vSpace). An example was provided in Section 7.7, where the addition of a visitor device into the Media vSpace triggers the evaluation of the security and privacy policy rules associated to the NAS device, resulting in the automatic disconnection of the NAS from this vSpace. However, to achieve further automation, it could be interesting to augment our solution with some capabilities such as device fingerprinting and device behavior monitoring. Fingerprinting would allow the controller to identify devices, their types and factory characteristics (e.g., the ones provided by the vendor including hardware and software characteristics). Monitoring would allow the controller to determine the behavior of the device connection patterns. As implementing those mechanisms might raise some scalability issues with the increase in the number of devices, some of our current work includes leveraging the network and compute power of the cloud to virtualize such functions. 

### 8.4. Ease-of-Use & Automation

In Section 6, we showed how automation and user-initiated network control are supported by the Future Spaces solution. Thanks to its end-to-end characteristics, network programmability and the mix of automation layers in the MajordHome component, the system enables much more complex levels of automation. For instance, we have prototyped a mechanism to automate guest device onboarding onto a vPlace. Using it, the vPlace owner can generate dedicated QR Codes which visitors can flash to be automatically connected to the FSGW with dedicated WiFi credentials and be put on a Visitors vSpace. The mechanism is implemented as an Assistant and leverages the control over a FSGW virtual network function service (i.e., WiFi authentication) as well as vSpace predicates. Another example is the possibility to rely on various network monitoring functions within the vPlace and Behaviors to make the vPlace react automatically to certain events (e.g., disconnecting a device trying to access a blacklisted URL in a security scenario or putting a device on a dedicated Multimedia vSpace when observing its UPnP/DLNA advertisement through monitoring). Finally, through external APIs of the Assistants, the system can ultimately serve as a substrate for smart home applications, by producing or reacting to broader context information, or even by hosting ambient intelligence logic.

While advanced automation provides for interesting scenarios, a potential challenge is its scalability in the face of frequent updates to the system. While the use cases investigated so far were for a large part related to user initiated actions (proper user requests to the system, or connecting devices to a FSGW), and so relatively infrequent and putting limited stress on the SDN-C and OVSes, fully autonomous scenarios triggering fast paced updates bring interesting stability questions, some of which require resorting to control theory and more traditional CPS solutions.

## 9. Conclusions

In this paper, we have presented Future Spaces, an end-to-end research solution prototype, which aims at giving users simple means to better control how their devices and home networks are used. Our solution relies on the dual concepts of Virtual Places, which manage users and connected resources of a possibly geographically distributed domain, and Virtual Spaces, which realize network slices whitelisting communication between authorized resources of one or several vPlaces. We presented our proof of concept prototype design and implementation, mixing a support infrastructure, a dedicated SDN controller and the accompanying vPlace resource manager and illustrating its usage in a scenario illustrating its salient features. The resulting end-to-end research testbed can serve as a sandbox for testing advanced security and networking scenarios for Smart Homes or even Enterprises, and we think some of its principles and mechanisms may be reused in other cyber-physical systems environments. We are in particular looking to apply it in 5G IoT scenarios, where different levels of Quality of Service can be requested to the underlying network infrastructure.
